# DNA-binding factor footprints and enhancer RNAs identify functional non-coding genetic variants

**DOI:** 10.1186/s13059-024-03352-1

**Published:** 2024-08-06

**Authors:** Simon C. Biddie, Giovanna Weykopf, Elizabeth F. Hird, Elias T. Friman, Wendy A. Bickmore

**Affiliations:** 1 MRC Human Genetics Unit, Institute of Genetics and Cancer, University of Edinburgh, Edinburgh, UK; 2 NHS Lothian, Edinburgh, UK

**Keywords:** Functional genetics, Functional genomics, Single nucleotide variants, Single nucleotide polymorphism, Non-coding genome, Non-coding variants, Genome-wide association study

## Abstract

**Background:**

Genome-wide association studies (GWAS) have revealed a multitude of candidate genetic variants affecting the risk of developing complex traits and diseases. However, the highlighted regions are typically in the non-coding genome, and uncovering the functional causative single nucleotide variants (SNVs) is challenging. Prioritization of variants is commonly based on genomic annotation with markers of active regulatory elements, but current approaches still poorly predict functional variants. To address this, we systematically analyze six markers of active regulatory elements for their ability to identify functional variants.

**Results:**

We benchmark against molecular quantitative trait loci (molQTL) from assays of regulatory element activity that identify allelic effects on DNA-binding factor occupancy, reporter assay expression, and chromatin accessibility. We identify the combination of DNase footprints and divergent enhancer RNA (eRNA) as markers for functional variants. This signature provides high precision, but with a trade-off of low recall, thus substantially reducing candidate variant sets to prioritize variants for functional validation. We present this as a framework called FINDER—Functional SNV IdeNtification using DNase footprints and eRNA.

**Conclusions:**

We demonstrate the utility to prioritize variants using leukocyte count trait and analyze variants in linkage disequilibrium with a lead variant to predict a functional variant in asthma. Our findings have implications for prioritizing variants from GWAS, in development of predictive scoring algorithms, and for functionally informed fine mapping approaches.

**Supplementary Information:**

The online version contains supplementary material available at 10.1186/s13059-024-03352-1.

## Background

Only a very small (2%) proportion of the human genome codes for protein-coding genes. Yet, genetic variants in the non-coding genome are known to contribute to Mendelian diseases and complex traits. Genome-wide association studies (GWAS) have uncovered thousands of genetic variants that are associated with traits or alter disease risk, and these loci predominantly lie in the non-coding genome [1] Identifying functional and causal non-coding variants from GWAS, and the target genes they regulate, remains a major challenge. The model for effects on trait proposes that non-coding variants alter the targets of sequence-specific nucleic acid binding proteins in cis-regulatory elements (CREs) resulting in quantitative changes in gene expression. This could be at the level of modulating RNA splicing by RNA binding proteins [2], or through altering the affinity for DNA binding proteins such as transcription factors (TF) at enhancers [3, 4]. Rare, pathogenic mutations in enhancers have been shown to reduce [5] or increase [6] TF binding. Contrasting the large functional affects for rare alleles, common single nucleotide variants (SNVs) tagged in GWAS are thought to subtly modulate TF occupancy at CREs [4, 7, 8].

Because GWAS identifies regions that contain multiple SNVs in linkage disequilibrium (LD), identifying the functionally relevant SNV(s) poses a significant challenge. One route is to analyze chromatin structure at active CREs. Binding of TFs at enhancers leads to nucleosome displacement, and recruitment of co-activators that can then further modify chromatin including, but not limited to, acetylation of histone H3 lysine 27 (H3K27ac) [9]. The Assay for Transposase-Accessible Chromatin using sequencing (ATAC-seq) is widely used for profiling sites with disrupted nucleosome structure and has been used in efforts to prioritize risk variants [10, 11]. Prior to the widespread use of ATAC-seq, DNase hypersensitive site-seq (DHS-seq) and DNase I footprinting have been used to identify sites of likely TF-binding and nucleosome disruption and to assess the effect of genetic variants on TF binding [4, 12, 13].

Enhancers and their target genes are most often located in the same topologically associating domain (TAD). Therefore, the integration of GWAS with data on tissue-specific features of active chromatin (ATAC-seq, DHS-seq, DNase footprinting, H3K27ac ChIP-seq) and chromosome conformation capture has been used to try and predict functional variants and their target genes [14, 15, 16]. However, predictions based on active regulatory marks often fall short in pinpointing functional variants [17, 18].

Computational models have also been developed to predict functional non-coding variants using machine learning methods including deep neural networks or linear regression models based on feature annotations, such as open chromatin, histone marks, TF binding, and conservation scores [19]. However, while showing some efficacy for rare variants, they poorly predict variant effects for common non-coding variants [19, 20]. Prioritizing and identifying functional non-coding variants therefore remain a barrier to GWAS follow-up studies.

Another feature of active enhancers is the production of short, unstable bidirectional enhancer RNAs (eRNAs) [21]. To the best of our knowledge, this feature has not been used in the prediction of functional genetic variants. Here, we analyze the utility of active regulatory element marks—open chromatin, H3K27ac, DNase footprints, TF binding, as well as the presence of divergent eRNA, for the prioritization and discovery of functional variants. We analyzed active regulatory markers using > 50,000 datasets from > 2000 cell types, tissue types, and treatment conditions and benchmarked these features with variants that demonstrate enhancer modulating activity using *cis*-acting molecular quantitative trait loci (molQTL). We find that the combination of DNase footprints and eRNA achieves high precision in predicting functional non-coding variants, and we apply this approach to data from GWAS traits. In this way, we demonstrate a framework to allow prioritization of candidate variants from GWAS data to aid discovery of functional and causative genetic variants.

## Results

### A compendium of genomic markers of active regulatory regions

**Fig. 1:**
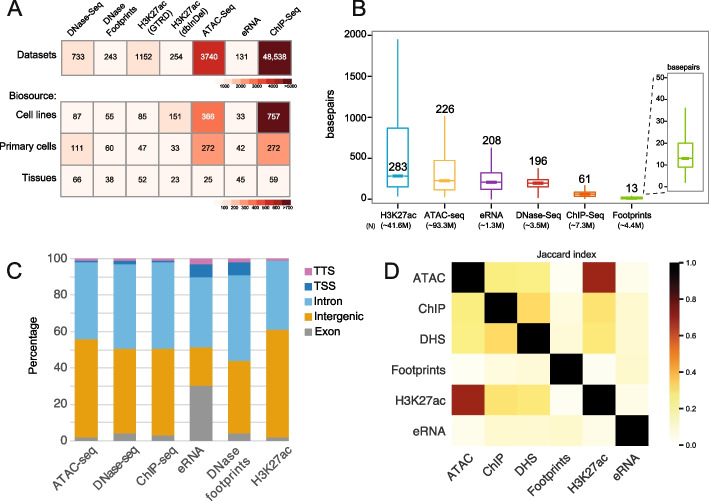
A compendium of markers of active regulatory regions. **A** Heatmap for six datasets that mark active regulatory elements. H3K27ac data were obtained from two different sources. Footprints are derived from DNase-seq data. The number of biosources (cell lines, primary cells, or tissues) are indicated. For ChIP-seq datasets, this consists of 956 DNA-binding factors. **B** Boxplot for each feature length from each biosource with the number shown indicating the median length in base pairs (bp). *N*, total number of elements for each feature, summed for all biosources regardless of overlapping features. **C** Genomic annotations for markers of active regulatory markers from merged biosources. TTS, transcription termination site; TSS, transcription start site. **D** Pairwise Jaccard index for merged intervals from DNase-seq, DNase footprints, ATAC-seq, ChIP-seq, eRNA, and H3K27ac

Functional non-coding genetic variants are thought to reside in CREs. Based on this, we collated a compendium of published datasets of markers for active CREs from human cells and tissues. We included markers for open chromatin (DHS from DNase-seq and Assay for Transposase-Accessible Chromatin (ATAC-seq)), H3K27ac, divergent enhancer RNA (eRNA), DNase footprints, and DNA-binding factor occupancy from chromatin immunoprecipitation (ChIP). eRNA data come from multiple assays including Cap Analysis Gene Expression (CAGE), Global run-on (GRO)-seq, Precision nuclear run-on (PRO)-seq, and capped small RNA-seq [22]. Data were collected from uniformly processed resources, which included multiple replicates, across cell lines, primary cells, or tissue biosources. In aggregate, this comprised > 50,000 datasets from > 2000 biosources (Fig. 1A, Additional file 1: Table S1). Most datasets are from ChIP-seq, including ChIP for 956 DNA-binding factors and for some factors across multiple cell types or treatment conditions. Only one cell-type, hepatocytes, shares data across all assay types (Additional file 1: Fig. S1).

Taking the aggregate across all biosources for each marker, the number of elements ranges from 1.3 million (eRNA) to 93.3 million (ATAC-seq), with the median length of the elements dependent on the assay (Fig. 1B). The majority of active CREs are found in the non-coding regions—intergenic regions, promoters, or introns (Fig. 1C). We computed the pairwise Jaccard index for markers, which shows the highest similarity is between ATAC and H3K27ac (Fig. 1D).

For each marker, we merged overlapping datasets across all biosources to determine the non-redundant genome coverage of active CREs. The largest coverage is represented by ATAC-seq (75.2%), followed by H3K27ac (61.5%). This is likely due to the high number of biosources (Fig. 1A), the longer median length and distribution (Fig. 1B), but also probably indicates too low a threshold used in peak calling. The smallest coverage is from eRNA (2.1%) followed by DNase footprints (3.6%) (Additional file 1: Fig. S1B).

### Prioritization of genetic variants by combinations of regulatory markers

**Fig. 2:**
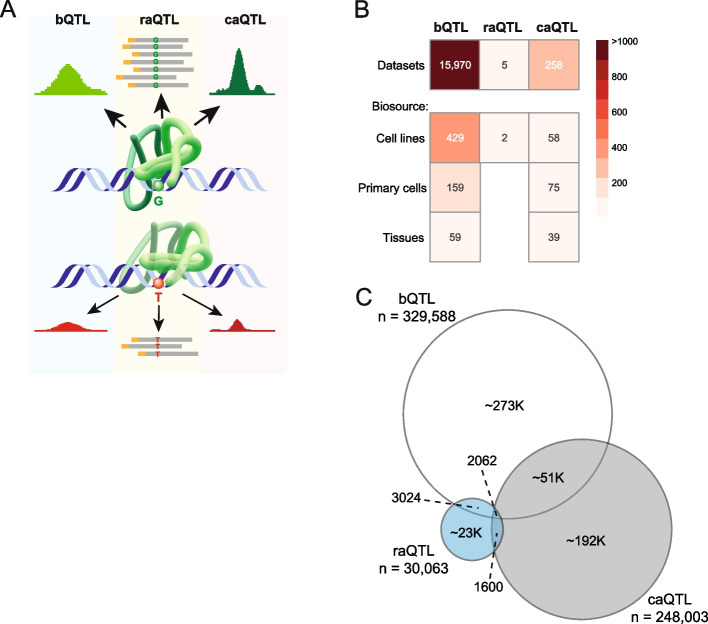
Molecular quantitative trait loci (molQTL) with effects on regulatory element activity. **A** MolQTL with effects on regulatory element activity include binding QTLs (bQTL), reporter assay QTLs (raQTL) from massively parallel reporter assays (MPRA), and chromatin accessibility QTLs (caQTL). The model depicts binding of a DNA-binding factor, which is altered by a single nucleotide variation, thus reducing binding, reporter assay expression, and chromatin accessibility. **B** Heatmap showing the molQTL datasets and biosources. bQTL data are derived from ChIP-seq experiments of 1073 DNA-binding factors. **C** Proportional Venn diagram of the number of QTLs from each assay showing minimal overlap

Functional non-coding genetic variants are likely to impact target gene expression through altering CRE activity. To assess the utility of markers of active CREs in prioritizing functional non-coding variants, we established a set of variants which colocalize with molecular quantitative trait loci (molQTL) to benchmark against. While genetic variants that impart phenotypic variation have numerous terms dependent on the molecular trait, we use the unifying term *QTL* here to include molQTL detected by regression, but also variants with allelic imbalance determined by other statistical methods. We selected three molQTL assays which measure different molecular mechanisms and which demonstrate variant effects in *cis* that impact CRE activity: DNA-binding factor QTL (bQTL) [23], massively parallel reporter assay (MPRA) QTL (raQTL) [24], and chromatin accessibility QTL (caQTL)—a merge of two sources [25, 26] and [13, 27]—also called chromatin-altering variants (CAV) (Fig. 2A). Variants identified by these molQTL assays are derived from > 16 K datasets across > 800 biosources (Fig. 2B, Additional file 1: Table S3). Most of these datasets are from bQTL assays based on ChIP-seq annotations, while caQTL are based on DNase-seq and ATAC-seq datasets. raQTL variants were obtained from one resource which identified raQTLs in K562 and HepG2 cell lines, using an unbiased set of 5.9 million variants [24]. We have not included other studies using MPRA to identify raQTL as these often use variants identified from specific GWAS traits which would bias our downstream analysis. There was minimal overlap between variants colocalizing with bQTL, raQTL, and caQTL (Fig. 2C). This maintains each molQTL assay as a largely independent set of variants.

Using each molQTL to benchmark, we aimed to identify functional variants from the GWAS catalog [28, 29], which is derived from > 28 K studies and > 5 K traits or diseases, and containing variant-trait associations with *p* value of ≤ 1.0 × 10^−5^ (Additional file 1: Table S3). This set contains > 180 K non-redundant variants, the majority of which are in non-coding regions (Additional file 1: Fig. S2A). To identify how active regulatory marks can functionally annotate non-coding variants, we merged all biosources for each mark, and in the case of ChIP this included merging all DNA-binding factors (Additional file 1: Fig. S2B). This overcomes two main issues. Firstly, that the cell- or tissue-type in which a variant is functional is not known a priori. Secondly, the low overlap of biosources for each marker (Fig. 1B) would limit intersection analysis. We therefore took a cell/tissue agnostic approach by merging biosources. With this, we determined the co-localization of the GWAS catalog variants with each marker of active CREs, and all combinations of markers, totaling 55 combinations from two-way up to six-way combinations.

**Fig. 3:**
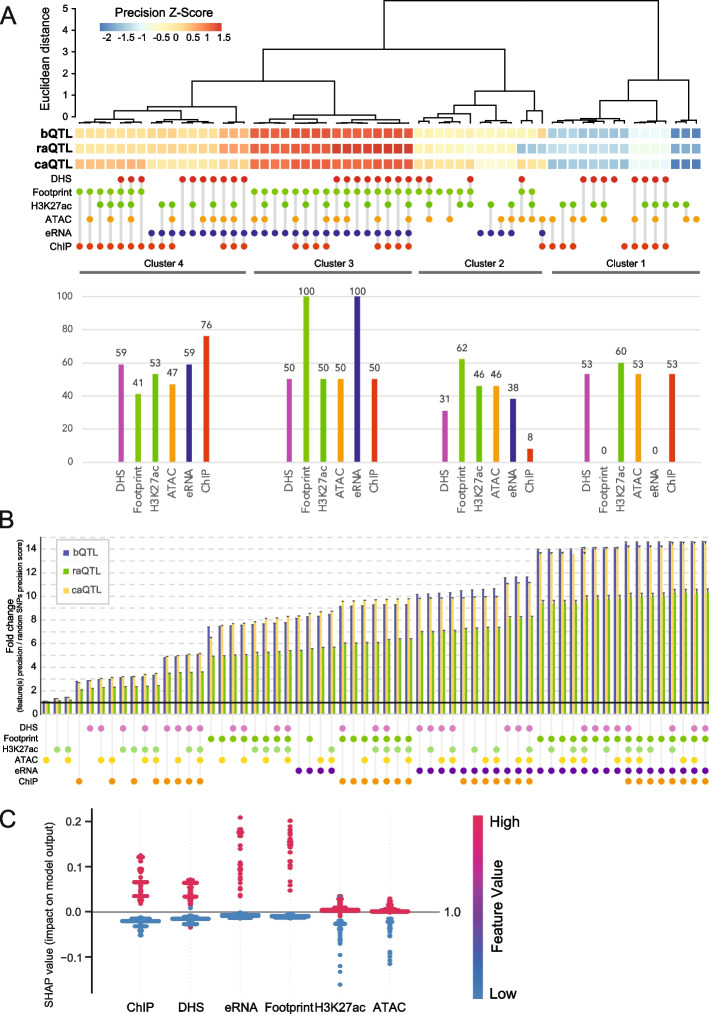
Identifying functional variants using combinations of markers of active regulatory elements. **A** Heatmap of precision *Z*-scores for individual, or combinations of, markers for GWAS catalog variants. Variants from the GWAS catalog were intersected with each feature or combinations of features, benchmarked against molQTLs. Unsupervised clustering was performed and percentages of each feature represented in each cluster are shown below. **B** Fold change of precision scores for functional variant discovery by feature combinations from random genomic SNVs. Common (MAF ≥ 1%) SNVs from the whole genome were subsetted to a comparable number to the GWAS catalog. Precision scores for variants that intersect with feature(s), and co-localize with a molQTL, are expressed as fold change relative to the number of subsetted variants that co-localize with molQTL. Feature combinations are sorted from lowest to highest fold change. One hundred iterations of subsetting from dbSNP were performed and error bars shows standard deviation. **C** SHAP (SHapley Additive exPlanations) values for a machine learning model using a random forest classifier to identify feature importance for each active regulatory mark towards the functional variant predictive model

We determined the precision (positive predictive value (PPV) − [true positive/(true + false positives)]) for each marker, and their combinations, by the intersection with variants from the GWAS catalog, benchmarked against the intersection of the marker combinations with each molQTL as a true positive set. The precision value was dependent on the size of the molQTL set relative to the larger GWAS catalog. The range of precision values was 7.5–55.46% for bQTL, raQTL 0.42–3.53%, and caQTL 4.02–27.7%. In all cases, the highest precision was achieved by the combination of all six markers. To compare precision values between the molQTL, we converted precision values to *Z*-scores for each molQTL, giving a range of scores from − 2.2 to 1.44. Using unsupervised clustering, we generated a *Z*-score heatmap of precision values for feature combinations against molQTL assays, showing four clusters (Fig. 3A). The cluster with the highest *Z*-scores (cluster 3) showed that two markers, DNase footprints and eRNA, were present for all feature combinations within the cluster, including the combination of these two markers alone. In contrast, the cluster with the lowest *Z*-score (cluster 1) excluded any combination of markers that had footprints or eRNA. As independent markers, DNase footprint or eRNA were present in an intermediate cluster (cluster 2). This suggests that the combination of DNase footprints and eRNA, with or without other markers, provides high precision for identifying functional variants. Increasing the number of features increased the number of predicted negative variants, although the number of predicted negative variants is > 90% for footprints or eRNA alone (Additional file 1: Fig. S3A).

While all six features are markers of active CREs, we considered if GWAS variants that intersect these features are associated with functional enhancers. Using a validated set of functional enhancers from CRISPR deletion and CRISPR interference experiments, and non-enhancers from MPRA experiments [22], we overlapped GWAS variants with feature(s). We find that GWAS variants in footprints and eRNA independently are associated with functional enhancers compared to other features alone (Additional file 1: Fig. S3B). However, GWAS variants that coincide with footprints and eRNA together have the highest overlap with functional enhancers.

We next considered the precision of marker combinations to identify functional variants across the entire genome, without association with GWAS traits. Taking all common genetic variants (minor allele frequency (MAF) ≥ 1%), consisting of > 660 M non-redundant SNVs, we randomly selected subsets to obtain a comparable number to the GWAS catalog of ~ 180 K variants. We calculated the precision of combinations of markers and then converted this to a fold change over random SNVs. We repeated the random SNV subsetting 100 times to generate a mean fold change and plotted the markers and combinations from lowest to highest fold change (Fig. 3B). This showed that ATAC-seq, H3K27ac, and the combination of the two are poor discriminators of functional variants (mean fold change 1.12–1.44). In comparison, a step change is observed for the combination of DNase footprints and eRNA, with a mean fold change of 13.89 over random SNVs. Footprints and eRNA alone show a mean fold change of 7.37 and 8.09, respectively, suggesting that they can individually provide functional annotation of variants across the genome, but with higher precision when combined. This is in keeping with the precision observed from footprints with eRNA to identify functional variants in the GWAS catalog. Our analyses suggest that the combination of DNase footprints and eRNA are strong predictors of functional variants with high precision, with additional markers providing only small increments in precision. Importantly, when considering the precision of GWAS compared to random genomic variants, the GWAS catalog shows a mean fold change of 8.24 which, perhaps expectedly, demonstrates that GWAS can identify functional variants, above a random model.

To corroborate our findings, we employed an independent machine learning approach. We trained a random forest classifier model to predict any molQTL signature (bQTL/raQTL/caQTL) based on overlaps with the six active regulatory markers, providing a predictive value of 0.64. We determined SHAP values as a measure of feature importance for the model, which revealed that DNase footprints and eRNA are the top positively associated features for the predictive model (Fig. 3C).

### Effect of thresholding of feature calls on precision scores

The local genomic enrichment of assay signals is often translated to interval regions (peaks) through different methods and at varying significance thresholds. These statistical variations in calling enriched regions, for example, false discovery rates (FDR), may impact precision scores for functional variants. For DNase footprints, we used consensus footprints determined by a posterior probability > 0.99 across biosources [13, 27]. We compared this with FDR for footprints from individual biosources and determined the precision scores associated with increasing FDR stringency. For footprints, we find that increasing stringency does increase precision, although the consensus footprint calling performs well (Additional file 1: Fig. S3C). For the remainder of our analyses, we restrict to the consensus set of footprints as these are derived across biosources and are less biased towards individual datasets or cell/tissue types. As ATAC-seq performs relatively poorly with regard to predicting functional variants, peak calling thresholds may impact precision scores. We used an independently analyzed source (ATACdb) [30, 31], which has a smaller number of datasets (~ 1400) compared to the ~ 3700 from GTRD [32, 33]. We further threshold these datasets by *p* value to increase stringency over local background. We find ATACdb to have a high precision score, but increasing *p* value thresholds does not improve precision scores. We have taken a third source of ATAC-seq data from ENCODE, using narrow peaks to define local regions of high amplitude and consider this dataset with and without irreproducible discovery rate (IDR) thresholding. We find the ENCODE narrow peaks precision scores improve slightly with IDR thresholding (Additional file 1: Fig. S3). Data processing and significance stringency can therefore impact the precision scores for identifying functional variants. However, this analysis shows that the relatively low predictive value of ATAC-seq peaks is not simply due to insufficiently stringent thresholding.

### Centrality in open chromatin to predict functional non-coding variants

**Fig. 4:**
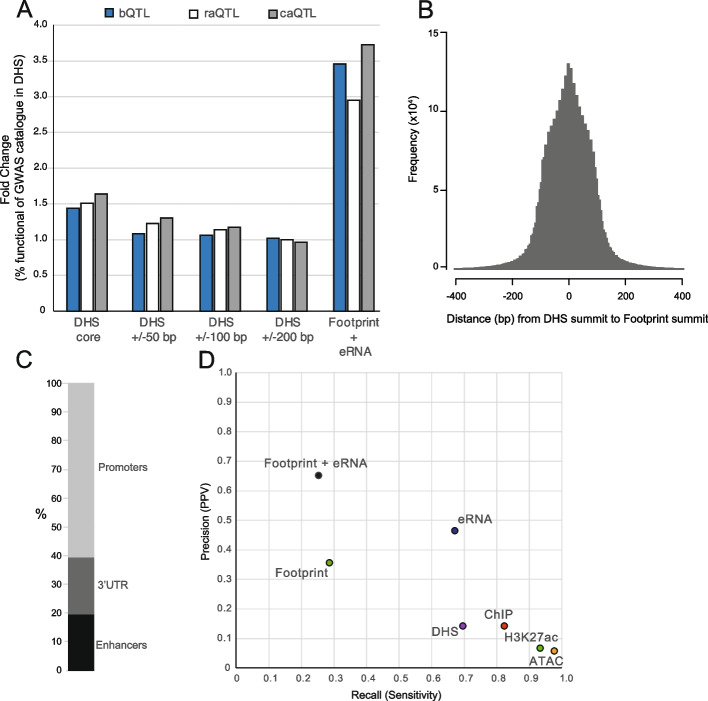
Centrality in the identification of functional variations, and benchmarking against non-coding Mendelian disease-associated variants. **A** Precision score of DHS cores and DHS central lengths around summits, compared to DNase footprints with eRNA. Precision scores are expressed as fold change of precision score for the indicated feature over the precision score for GWAS variants alone. Scores are benchmarked for each molQTL. **B** Distribution of nearest DHS footprint up- and downstream relative to DHS summits. **C** Genomic annotation of validated, rare, non-coding Mendelian disease-associated variants. **D** Precision-recall graph determined from 230 non-coding Mendelian disease-associated variants, with spike-in of random genomic variants from dbSNP (~ 4 K) to produce a validated variant probability of ~ 0.05. Features were intersected to determine precision (positive predictive value—PPV) and recall (sensitivity). Binary outcomes were determined from feature intersections

TF binding can initiate and maintain chromatin accessibility [34] and indeed summits of DHS have been shown to be enriched for DNA-binding factor motifs [13]. Using a consensus reference map of DHS with summit annotations [35, 36], we tested if centrality in DHS could predict functional variants. We compared core DHS regions, which comprises a consensus centroid region aggregated across biosamples, and distances from the summit at 25 bp, 50 bp, and 100 bp up- and downstream, with a median core length of 37 bp (Additional file 1: Fig. S4A, B). By using variants from the intersection with the GWAS catalog benchmarked against molQTL variants, we calculated precision scores as fold change above GWAS catalog alone. DHS cores have ~ 1.5-fold precision, and this is not increased by extending to 100 bp on either side of the summit (Fig. 4A). Although many footprints fall outside the DHS core, DNase footprints are found at a higher frequency nearer DHS summits (Fig. 4B). Together, these data suggest a mechanism for functional variants related to the enrichment of DNA-binding motifs near DHS summits. When comparing centrality as a means to identify functional variants with DNase footprints and eRNA, the precision is even higher with the combined DNase footprints/eRNA markers (Fig. 4A).

### Replication with functional, rare, non-coding Mendelian disease-associated variants

To validate our observation of high precision using the combination of DNase footprints and eRNA, we sought to replicate our analysis using a set of validated functional non-coding variants. We used a published set of manually curated rare non-coding variants associated with Mendelian diseases consisting of 210 non-redundant variants in promoter, intergenic, intronic, or 3′UTR regions [37] (Fig. 4C). Given the small number of variants within this validated set, we limited our precision calculations to single markers, or the combination of DNase footprints and eRNA only. We determined precision by spike in of ~ 4 K randomly selected variants across the whole genome, giving a true positive rate of approximately random chance (~ 0.05). We generated precision and recall scores based on the mean from 10 permutations of random genomic variant spike-ins (Fig. 4D). The combination of DNase footprints and eRNA shows the highest precision at 0.65, but low recall (0.25). In contrast, ATAC-seq and H3K27ac show low precision but high recall. DNase footprints and eRNA each alone have intermediate precision and recall. These observations are consistent with our analysis of the GWAS catalog and genomic variants, suggesting the utility of DHS footprints and eRNA in identifying both rare and common functional non-coding variants.

### Prioritizing variants from genome-wide association studies

**Fig. 5:**
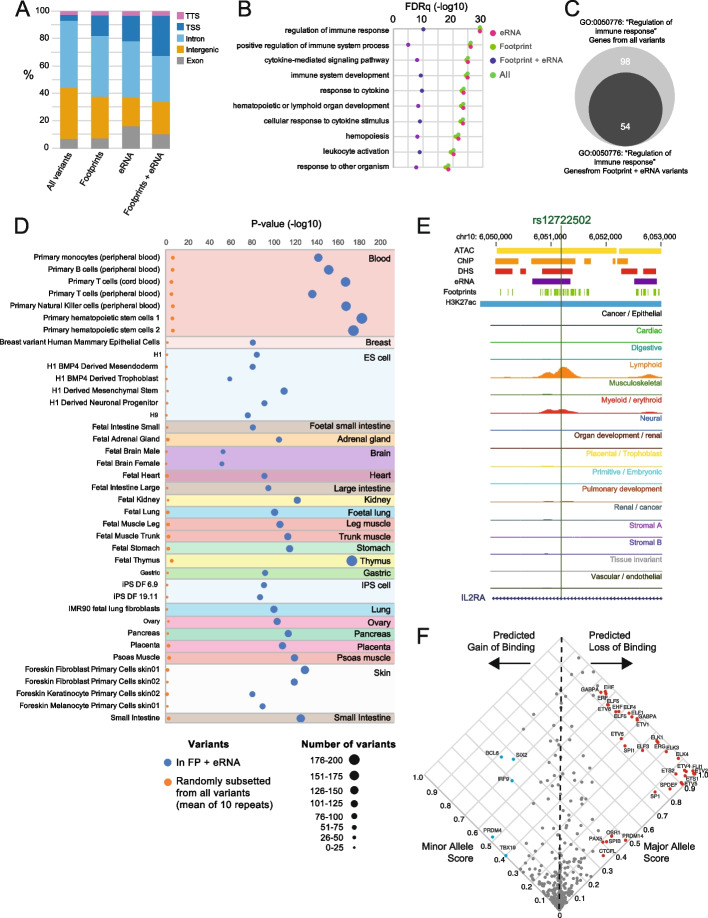
Prioritization of variants using DNase footprints and eRNA preserve cell-specific features and functions. **A** Genomic annotation of all variants for 53 traits from the GWAS catalog, and annotations when intersection with DNase footprints, eRNA, or both in combination. **B** Gene ontology (GO) analysis of proximity genes relative to variants of lymphocyte count trait, for all variants for the trait from the GWAS catalog, variants in DNase footprints, eRNA, or both. Significance of the GO term is expressed as − log10 of the FDR *q* value (FDRq). Top ten GO terms for all variants are shown with FDRq for each intersection. **C** Number of genes for the GO term “regulation of immune response” comparing number of genes from all variants compared to number of genes from variants in footprints and eRNA. **D** Enrichment analysis, using FORGE2, of cell-type specific active CREs for the lymphocyte count trait, comparing variants in DNase footprints and eRNA, compared to an equal number (333) of randomly selected variants from all lymphocyte count variants from the GWAS catalog. The random selection was repeated ten times, and the mean *p* value (− log10) is shown. **E** An example lymphocyte count-associated variant (rs12722502) at the *IL2RA* locus which co-localizes with a DNase footprint and eRNA. UCSC genome browser shot shows merged features and ENCODE DNase-seq bigwig tracks for each cell-/tissue-type. **F** Predicted impact of rs12722502 on DNA-binding factors. Scatter plot of the major and minor allele binding score for DNA-binding factor motifs showing predicted gain or loss of binding. Example DNA-binding factors are labeled

To demonstrate the utility of DNase footprints and eRNA in prioritizing non-coding variants, we analyze 53 traits for intersections with footprints, eRNA, or both (Table 1). Across these traits, the majority of associated variants are, as expected, in the non-coding genome, and this is maintained when using the combined footprint and eRNA marks to prioritize variants (Fig. 5A). Using the leukocyte count trait from the GWAS catalog to exemplify our prioritization approach, ~ 10 K non-redundant variants with significance of < 9 × 10^−6^ from 51 studies are reduced ~ 30-fold by requiring co-localization with DNase footprints and eRNA (Table 1). Using gene ontology (GO) analysis of neighboring genes, we applied GREAT analysis [38] to all variants from the trait compared to just the variants within footprints and eRNA. GO terms are preserved between all variants and those in footprints and eRNA (Fig. 5B), with enriched GO terms in keeping with leukocyte function. For the most significant GO term, regulation of immune response, we observe preservation of 55% of genes in the footprint and eRNA variant set, compared with all variants (Fig. 5C) suggesting that, despite a ~ 30-fold reduction in number of candidate variants, the enriched genes and loci are largely preserved.

**Table 1:** Sources for each type of dataset used. For more detail please see Additional file: Table S1

Dataset	Source	Reference
ATAC-seq	GTRD	[32, 33]
ATAC-seq	ATACdb	[30, 31]
ATAC-seq	ENCODE	[39, 40]
caQTL	QTLbase	[25, 26]
CAV	ENCODE	[13]
ChIP-Seq	GTRD	[32, 33]
bQTL	AD_ASTRA	[23, 41]
CRISPRdel_CRISPRi	Primary_paper	[22]
DHS	ENCODE	[35, 36]
eRNA	PINTS	[22, 42]
DNase footprints	ENCODE	[13, 27]
GWAS catalog	NHGRI-EBI	[28, 29]
H3K27ac_dbInDel	dbInDel	[43, 44]
H3K27ac_GTRD	GTRD	[32, 33]
raQTL_MPRA	Primary_paper	[24]

The PeakPredict package is available on GitHub under an MIT license at https://github.com/efriman/PeakPredict [45] and at Zenodo: https://zenodo.org/doi/10.5281/zenodo.12706471 [39]

Non-coding variants function in a cell-type specific manner (e.g., [46, 47]) but uncovering the affected cell-type from GWAS can be challenging. By leveraging DNase footprints and eRNA to identify functional variants with high precision, we can then infer the relevant cell-type from this subset of variants. As both footprints and eRNA are associated with open chromatin [25], we took the leukocyte count associated variants co-localizing with footprints and eRNA and identified cell-type specific enrichment of DHS using FORGE2 [48]. Since footprints are derived from DNase-seq, the subset of variants that colocalize with DNase footprints and eRNA show a greater enrichment in DHS compared to an equal number of randomly selected GWAS variants for leukocyte count. Importantly however, despite our cell- and tissue-type agnostic approach of merging features, colocalized variants show enrichment in hematopoietic or immune cell types (Fig. 5D), demonstrating preserved ability to identify trait relevant cell-types despite ~ 30-fold reduction in variant number. Highlighting one variant identified from DNase footprints and eRNA, rs12722502 is an *IL2RA* intronic variant and is in a myeloid and lymphoid specific DHS (Fig. 5E). This variant is predicted to cause a loss of binding of ETS family TFs (Fig. 5F), members of which are known to regulate immune cell function [49]. Indeed, rs12722502 is associated with fivefold reduction in binding of ELF1 (a TF in the ETS family), in a human leukemic T-cell line [50].

### Identifying functional variants in linkage disequilibrium with lead variants

**Fig. 6:**
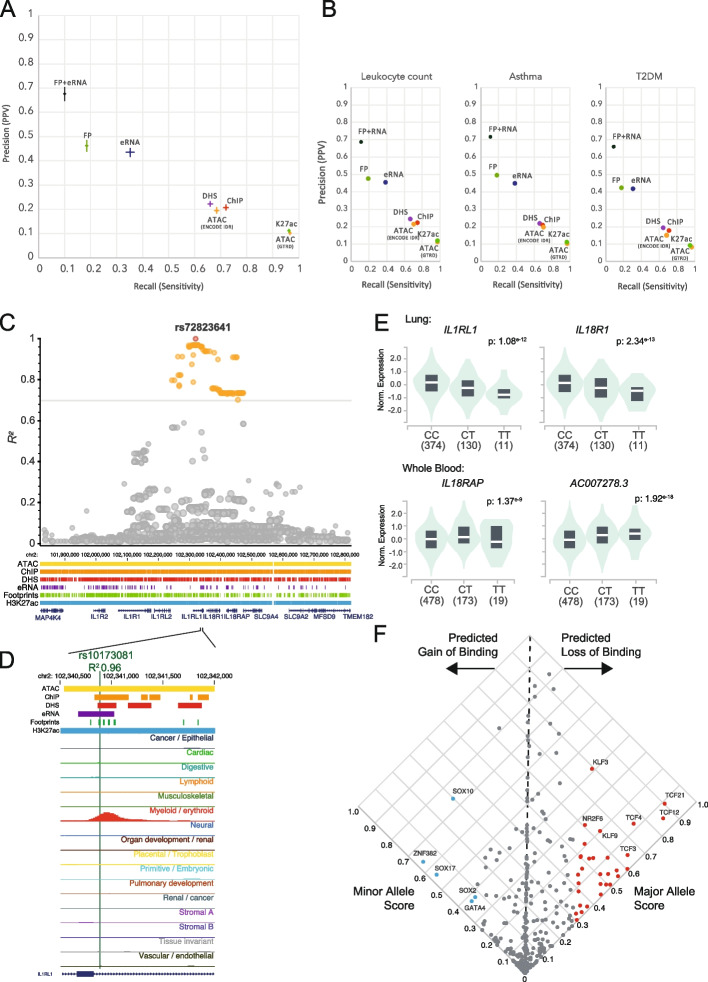
DNase footprints and eRNA can prioritize functional variants for variants in LD with a lead variant. **A** Using GWAS variants from 53 traits, mean precision and recall scores were determined by intersecting indicated feature(s) with GWAS and LD variants, against a combined set of QTL variants from bQTL, raQTL, and caQTL. LD variants were determined for each GWAS variant using an *R*^2^ > 0.7 For ATAC, intervals were obtained from GTRD, or ENCODE with IDR thresholding. Lines across the mean show the 95% confidence interval of the precision or recall score. **B** Precision and recall scores for GWAS and LD variants for the leukocyte count trait, asthma, and type 2 diabetes (T2DM). **C** Manhattan plot of the asthma-associated rs72823641 variant (red), with variants in LD ≥ 0.7 indicated in orange. Merged feature tracks are shown for the locus. Genome co-ordinates (Mb) are indicated. **D** rs10173081 overlaps a DNase footprint and eRNA. UCSC genome browser shot for the intronic region of *IL1RL1* with rs10173081 overlapping a myeloid specific DHS. **E** rs10173081 is an eQTL for multiple genes in lung and whole blood. GTex violin plots for normalize expression of the indicated genes across homozygous major allele, heterozygous major and minor allele, and homozygous minor allele. **F** Predicted impact of rs10173081 on DNA-binding factors. Scatter plot of the major and minor allele binding score for DNA-binding factor motifs showing predicted gain or loss of binding. Example DNA-binding factors are labeled

Uncovering functional or causative variants from GWAS is also challenged by LD, whereby the causative variant may be a variant in LD with the GWAS lead variant. We analyzed the utility of the six active regulatory features, and the combination of footprints and eRNA, to identify functional variants in LD with GWAS variants. Using GWAS variants from 53 traits, and variants in LD (*R*^2^ > 0.7) with each GWAS variant, we curated ~ 1.5 M variants across traits, which together consisted of ~ 800 K non-redundant variants. Taking GWAS and LD variants for each trait, we calculated precision and recall scores, by benchmarking against the combined set of variants in bQTL, raQTL, and caQTL. We determined mean precision and recall scores for the regulatory feature(s) (Fig. 6A, B) and observe the DNase footprint and eRNA combination to reproducibly have the highest precision, but low recall. While including variants in LD is important, this increases the variant set by a mean of ~ 27-fold across traits. However, considering only LD variants intersecting footprints and eRNA reduces the variant set by a mean of ~ 80-fold (Additional file 1: Table S2).

To demonstrate the validity of colocalization with DNase footprints and eRNA to prioritize variants in LD with lead variants, we selected the asthma trait, and considered strong lead GWAS variants which were not associated with the *HLA* locus, to avoid cell-type bias. We identified rs72823641, which has been found to be associated with asthma in multiple GWAS [51], with *p* values ranging from 4 × 10^−12^ to 2 × 10^−136^, and mapping to a large intron of *IL1RL1*. We found 92 variants in LD with rs72823641 (*R*^2^ > 0.7) using European ancestry data (Fig. 6C). Intersecting these with DNase footprints and eRNA reduced the variants to one candidate—rs10173081 (*R*^2^ = 0.96), which resides in myeloid specific open chromatin (Fig. 6D). While rs10173081 is not tagged in the GWAS catalog, it has been observed in other GWAS for asthma [52]. rs10173081 is also an eQTL for *IL1RL1* and *IL18R1* in lung, but also for *IL18RAP* and a non-coding RNA AC007278.3 in whole blood (Fig. 6E). Applying TF motif based binding prediction, we identified putative loss of binding for TFs including TCF21, TCF12, TCF4, and KLF3 (Fig. 6F). Interestingly, *TCF21* and *KLF3* loci have been previously identified as asthma risk-associated GWAS loci [53]. In conclusion, by using combined DNase footprint and eRNA markers, plausible functional variants can be prioritized from variants sets in LD with lead variants.

## Discussion

GWAS have been instrumental in uncovering complex trait- and disease-associated variants and regulatory pathways. However, the identification of functional and causative variants remains a significant challenge, impacting the mechanistic understanding of complex disease genetics and the identification of disease/trait relevant pharmacological targets. Consequently, few GWAS have been translated into therapeutic interventions [54]. Discovering functional variants in the non-coding genome constrained by methodological limitations. Computational approaches have been developed that utilize variable genomic features to aid variant prioritization, including functional annotations, conservation scores, and TF motifs [19, 55]. However, for non-coding variants, these methods tend to have low precision for identifying functional variants [19].

Here, we systematically analyzed six assays for active CREs, benchmarking against independent molQTLs for enhancer activity. We find that ATAC peaks and H3K27ac are poor predictors. This is in keeping with previous studies that have shown that deletion of elements harboring candidate variants marked by ATAC peaks or H3K27ac is unable to detect functional impact [17, 18, 56]. We hypothesize that the poor precision, but high recall, of ATAC-seq and H3K27ac peaks relates to their high feature size and high genomic coverage. In contrast, we identify a signature that performs well with respect to precision in identifying genetic variants with putative function. DNase footprints and eRNA have independently been suggested to be enriched for putative functional variants [13, 22]. Individually DNase footprints and eRNA both show good precision; however, we demonstrate that their combination provides superior discrimination. When using genomic features for prioritizing functional variants, consideration should be made for statistical stringency as this may alter precision for functional discovery. Additionally, centrality may contribute, but we find including central regions imparts little improvement in precision compared to the combined DNase footprint and eRNA marks.

Our model for the precision offered by combined DNase footprints and eRNA markers is based on direct DNA binding of a factor, such as a TF, leading to recruitment of RNA polymerase II and other machinery to promote regulatory activity. In agreement with this model, eRNAs have been shown be an independent predictor of functional enhancers in mice in vivo [57], and to predict TF binding, where TF binding and eRNA origins colocalize [4]. eRNAs have also been found to mark functional enhancer-promoter interactions [58]. Together, these suggest eRNAs are important markers of functional CREs.

Some markers of active elements perform with low precision, such as open chromatin and DNA-factor binding (ChIP). Consistent with this, < 30% of ATAC peaks in primary CD4 + T-cells demonstrated activity using an MPRA, with MPRA signals instead correlating well with eRNA production [59]. If evidence of binding is required for functional variant prediction, then ChIP-seq signals would be expected to perform well. However, MPRA of glucocorticoid receptor binding sites showed < 20% had significant activity [60], suggesting that many bound TFs are not productive. However, artefactual binding observed from cross-linking in ChIP-seq experiments [61], and co-localization of multiple TF binding events enriching for non-related motifs, can lead to false positive binding sites [62]. Therefore, the footprint signal of direct TF binding, with a detection of eRNAs, provides better evidence for an active enhancer. Variants within these regions, particularly in DNA sequences interfacing with TF DNA binding domains (DBD), are thus more likely to be functional.

Despite the high precision for functional variants using the combination of DNase footprint and eRNA, restricting to these two markers comes with limitations, principally related to low recall, and a high number of false negatives. The low recall can be explained by limitations inherent to the DNase footprint and eRNA datasets. Firstly, the identification of DNase footprints may be related to residence time of TFs on DNA, where rapidly exchanging factors impart poor footprints [63]. Variants associated with altering binding of dynamic factors may therefore be missed, although this could be improved using modified methods such as DNase-seq with crosslinking [64]. Detection of footprints could also be improved by bias correction to improve false-negative rates [65]. Secondly, the eRNA database used in our analysis represents the lowest number of biosources, and is therefore incomplete, with highly variable numbers of features between cell-types, and predominantly under basal conditions, which would exclude context-dependent enhancer activity [22, 60]. To address this, eRNA profiling is required in cell-types, and under conditions, relevant to the GWAS trait of interest. The specific assay for divergent eRNAs may also impact precision scores for functional variants, as GRO-cap has been reported to have the highest sensitivity for functional enhancer detection [22].

## Conclusion

**Fig. 7:**
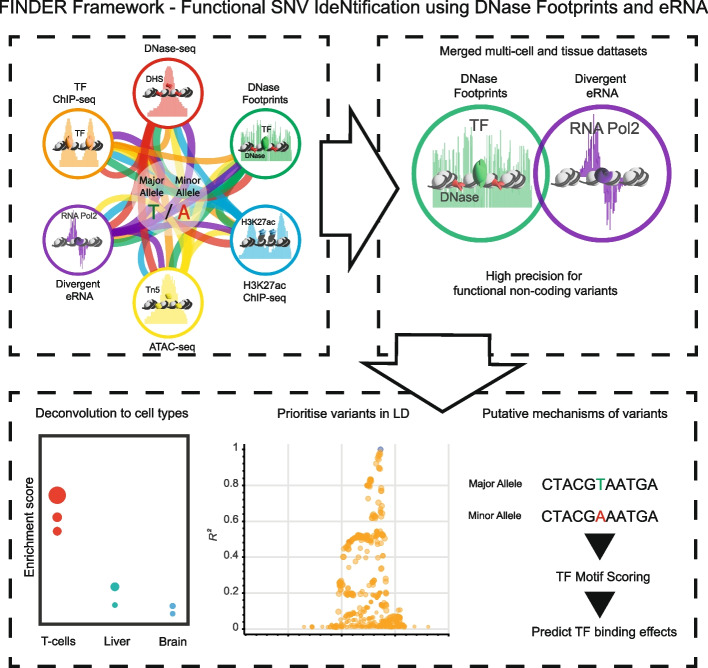
The FINDER framework—Functional SNV IdeNtification using DNase footprints and Enhancer RNA. Markers of active regulatory elements as merged datasets from multiple cell- and tissue-types are used to predict functional variants. The combination of DNase footprints and eRNA provides the highest precision, which can be applied to GWAS traits or to predict functional variants in linkage disequilibrium (LD). Predicted variants can then be interrogated for cellular and molecular function, by deconvolving marker enrichment to predict relevant cell-types, or the predict altered binding affinity for DNA-binding factors

We present our findings as a framework, FINDER—Functional SNV IdeNtification using DNase footprints and Enhancer RNA (Fig. 7). In this framework, the combination of DNase footprints and eRNA can prioritize and reduce candidate variants to a set with high precision. The merged datasets used can be analyzed for relevant cell-types associated with the complex trait or disease. This has important implications for current computational approaches and databases for predictive functional variant scores. Our findings can inform predictive scoring approaches as these do not utilize eRNA as a predictive marker. Finally, the high precision of DNase footprints and eRNA compared to other marks may be used for functionally informed fine mapping approaches [66], which could improve detection of causative non-coding variants.

## Methods

### Data sources

Datasets for active chromatin marks were obtained from published and publicly available sources. DHS [35, 36] and DNase footprints [13, 27] were obtained from ENCODE. H3K27ac was obtained from GTRD [32, 33] and dbInDel [43, 67]. Human ChIP-seq and ATAC-seq datasets were obtained from GTRD [32, 33]. Enhancer RNAs were obtained from the PINTS portal [22, 44]. These data sources provide uniformly analyzed datasets for downstream analysis. Details of downloaded versions and sources are shown in Additional file 1: Table S3. For each type of active chromatin mark, to generate assay master files agnostic to multiple cell-types or factor in the case of ChIP, Bedtools merge version from the bedtools suite v2.30.0 [42] was used, requiring overlap of 1 bp or greater. The Jaccard index was computed using bedtools *jaccard* from the bedtools suite v2.30.0 [42].

QTL datasets were obtained from published datasets. Binding QTL (bQTL) were downloaded from AD ASTRA which determines allele-specific binding events from human ChIP-seq datasets and identifies genetic variants from ChIP-seq data [23, 68]. Reporter assay QTL (raQTL) data were downloaded from publicly available massively parallel reporter assays (MPRA) [24]. Chromatin accessibility QTL (caQTL) were downloaded from ENCODE [13, 27] and QTLbase [25, 26]. Details of downloaded versions and sources are shown in Additional file 1: Table S3. For each molecular QTL, where an rsID occurs in multiple datasets, either multiple assays (such as ChIP) or multiple cell types, these were collapsed to generate a list of unique rsIDs.

GWAS summary statistics were downloaded from the NHGRI-EBI GWAS catalog V1.0.2 [28, 29] using variants with a *p* value < 10^−5^. Where analysis presented is agnostic to traits or studies, a master list of rsID found in the GWAS catalog was generated by collapsing to a list of unique rsIDs. Details of downloaded version and source are shown in Additional file 1: Table S3.

The dataset for genetic variants was obtained from UCSC [41] using the dbSNP build 151 containing uniquely mapping common SNPs with a ≥ 1% minor allele frequency (MAF). SNPs were randomly subsetted using gshuf in command line.

### Confusion matrix calculations and normalizations

Precision scores were calculated as the fraction of the number of variants (GWAS catalog or other variant sets as indicated) overlapping the benchmarked molQTL (true positives) over the total number of variants (true and false positives) intersecting the feature (or feature combinations). Precision scores were expressed as a *Z*-score or fold change. Clustering of precision *z*-scores was performed in R using heatmap cluster function with the Ward D2 method. Fold changes were expressed as the precision score of the candidate set intersected with features divided by precision score of the candidate set without intersection with features.

### Genome build conversions

Data were analyzed using human genome build Hg38. Where primary data sources were aligned to other genome versions (Hg 19), data was converted to Hg38 using Liftover [69]. The genome version of the primary data sources is indicated in Additional file 1: Table S3.

### Intersections of features

To analyze the overlap between genetic variants, and/or genomic features, Bedtools intersect from the bedtools suite v2.30.0 [42] was used. For intersections with rsID, these were considered as the first feature (-a) in bedtools intersect -a < bed/vcf > -b < bed > .

### Random forest classification

The PeakPredict package [45] was run using the command overlap_peaks with settings “–predict_column molQTL –model RandomForestClassifier –balance –shap,” where balancing downsamples the groups to have the same size prior to splitting into test and training sets. The PeakPredict package implements scikit-learn [70] and SHAP values [71].

### Gene ontology analysis

GO analysis utilized Genomic Regions Enrichment of Annotations Tool (GREAT) version 4.0 [38] with rsID converted to BED format, using the default settings.

### Predicting functional base on motif

Predictions of TF binding based on motifs utilized the FABIAN-variant [72] web interface, using Hg38m, using “All” TFS, the “TFFM detailed” model, and the JASPAR2022 database.

### Linkage disequilibrium analysis

To generate variants in LD with a lead variant, we included variants within 500 Kb of a lead variant with an *R*^2^ ≥ 0.7 based on European ancestry and a minor allele frequency of at least 1%. To compile variants in LD, we used TopLD API with options -thres 0.7 -pop EUR -maf 0.01 [73].

### Cell-type specific analysis

To identify cell-type enrichment of variants, we used FORGE2 [48], with input as rsID of variants, using DHS as data for enrichment, without LD filtering of rsIDs.

### Genome location mapping

To annotate the genomic features to genome location, we used the annotatePeaks.pI program from HOMER tools v4.1 [74]. Gene annotation was customized using a GTF file containing hg38 gene transcript sets, downloaded from UCSC (hgdownload.soe.ucsc.edu/goldenPath/hg38/bigZips/genes/hg38.refGene.gtf.gz)

### Supplementary Information

Additional file 1:  Supplementary figures and tables.

Additional file 2. Review history.

## Data Availability

Individual data sets are described in “Data sources” of the “Methods” section. The merged datasets for each active regulatory marker, merged as described in “Data sources,” and QTL merged datasets can be found at GitHub [77], available under an open source (MIT) license, and at Zenodo [78]. Further details are found in Additional file 1: Table S3.
