# Supplementary material for: DNA-binding factor footprints and enhancer RNAs identify functional non-coding genetic variants
Source: Genome Biol. 2024 Aug 6;25:208. doi: 10.1186/s13059-024-03352-1 (PMC11304670; doi:10.1186/s13059-024-03352-1)
Supplement: Supplementary file 1 — Additional file 1: Supplementary figures and tables. [file 13059_2024_3352_MOESM1_ESM.pdf]

Figure S1

A

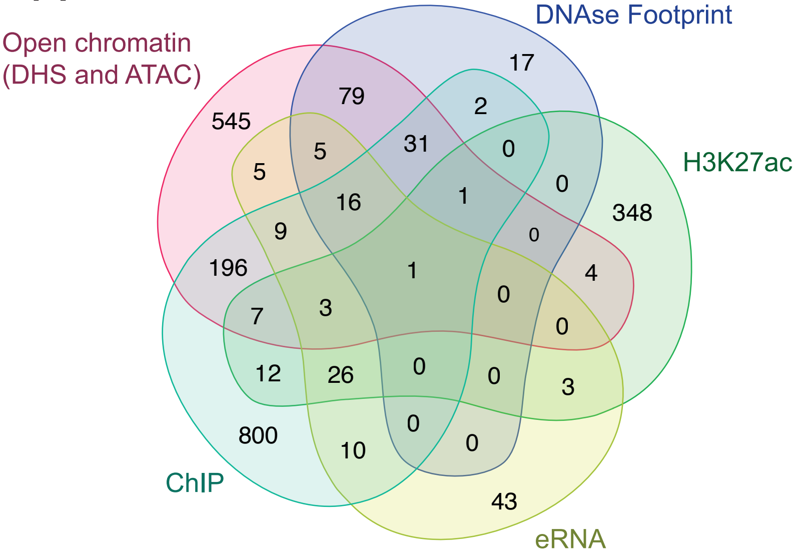
Open chromatin (DHS and ATAC)
DNAse Footprint
H3K27ac
ChIP
eRNA
545
79
17
2
5
5
31
0
0
16
1
348
9
0
196
1
4
0
3
7
0
12
26
0
0
3
800
0
10
0
43

B

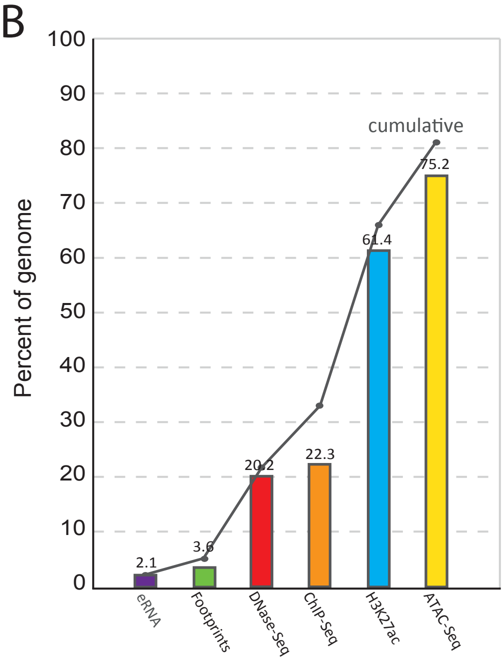
Percent of genome
100
90
80
70
60
50
40
30
20
10
0
cumulative
75.2
61.4
22.3
20.2
3.6
2.1
eRNA
Footprints
DNase-Seq
ChIP-Seq
H3K27ac
ATAC-Seq

Figure S1. Features of markers of active regulatory elements.

A. Euler diagram for intersections of biosources for each type of feature. DNase-seq (DHS) and ATAC-seq have been combined for visualisation.

B. Percentage of genome coverage of total number of genomic basepairs (Hg38 build), with the percentage for each feature indicated. The line graph indicates the cumulative percentage.

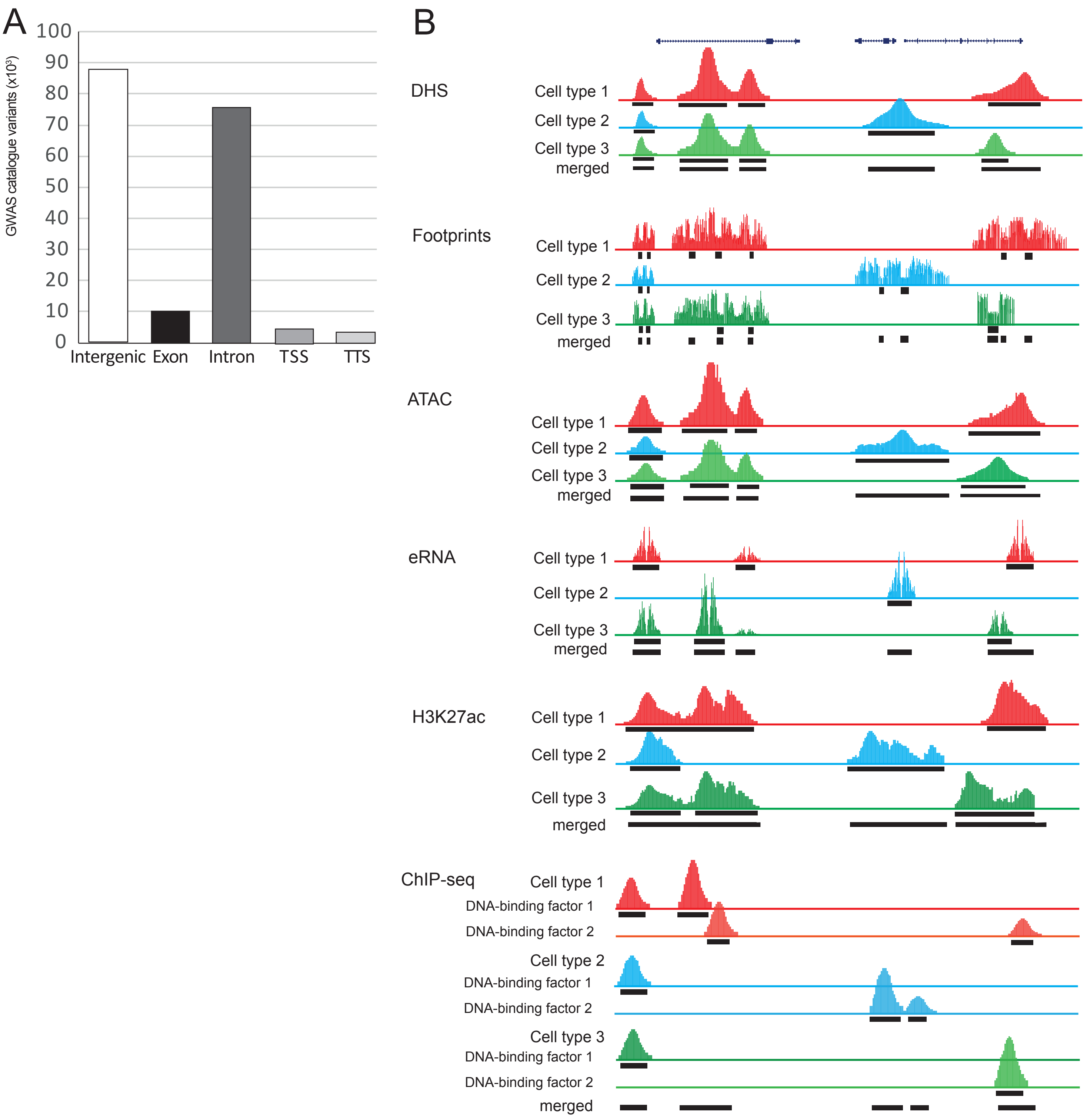
A
GWAS catalogue variants (x10³)
100
90
80
70
60
50
40
30
20
10
0
Intergenic
Exon
Intron
TSS
TTS
B
DHS
Cell type 1
Cell type 2
Cell type 3
merged
Footprints
Cell type 1
Cell type 2
Cell type 3
merged
ATAC
Cell type 1
Cell type 2
Cell type 3
merged
eRNA
Cell type 1
Cell type 2
Cell type 3
merged
H3K27ac
Cell type 1
Cell type 2
Cell type 3
merged
ChIP-seq
Cell type 1
DNA-binding factor 1
DNA-binding factor 2
Cell type 2
DNA-binding factor 1
DNA-binding factor 2
Cell type 3
DNA-binding factor 1
DNA-binding factor 2
merged

Figure S2

Figure S2. Genomic features of the GWAS catalogue and generation of merged active regulatory element datasets.

A. Genomic annotation of variants from the GWAS catalogue showing that the majority reside in the non-coding genome. TSS; transcription start sites, TTS; transcription termination site.

B. Illustrated examples of merging of different biosources for the same feature to generate a cell- and tissue-agnostic set.

Figure S3

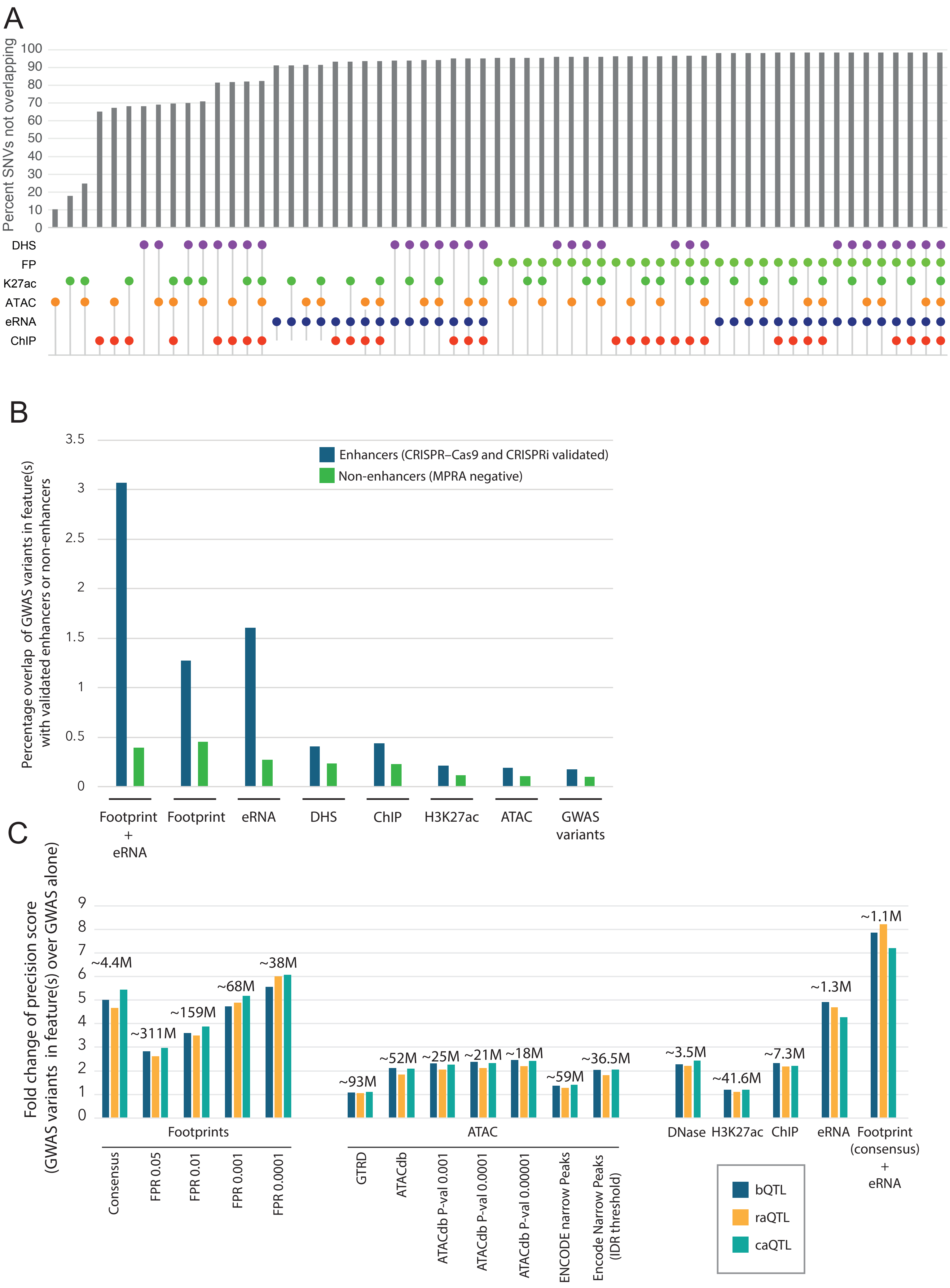
A
Percent SNVs not overlapping
0
10
20
30
40
50
60
70
80
90
100
DHS
FP
K27ac
ATAC
eRNA
ChIP
B
Percentage overlap of GWAS variants in feature(s) with validated enhancers or non-enhancers
Enhancers (CRISPR–Cas9 and CRISPRi validated)
Non-enhancers (MPRA negative)
0
0.5
1
1.5
2
2.5
3
3.5
Footprint + eRNA
Footprint
eRNA
DHS
ChIP
H3K27ac
ATAC
GWAS variants
C
Fold change of precision score (GWAS variants in feature(s) over GWAS alone)
0
1
2
3
4
5
6
7
8
9
~4.4M
~311M
~159M
~68M
~38M
~93M
~52M
~25M
~21M
~18M
~59M
~36.5M
~3.5M
~41.6M
~7.3M
~1.3M
~1.1M
Footprints
ATAC
DNase
H3K27ac
ChIP
eRNA
Footprint (consensus) + eRNA
Consensus
FPR 0.05
FPR 0.01
FPR 0.001
FPR 0.0001
GTRD
ATACdb
ATACdb P-val 0.001
ATACdb P-val 0.0001
ATACdb P-val 0.00001
ENCODE narrow Peaks
Encode Narrow Peaks (IDR threshold)
bQTL
raQTL
caQTL

Figure S3. Characteristics of features as predictors of functional variants

A**.** Percentage of GWAS catalogue (~181K) variants which are exluded from overlap with individual feature, and feature combinations, classed as predicted negative sets.B. GWAS catalogue variants that overlap with feature(s), or alone, were intersected with a set of validated enhancers from CRISPR deletion (CRISPR/Cas9) and CRISPR interference (CRISPRi) experiments, and non-enhancers from MPRA (70).

C. Precision scores for GWAS variants overlapping feature(s) for bQTL, raQTL and caQTL are normalised as fold change over GWAS variants alone. Different FDR thresholds for DNase footprints were compared to the consensus footprint dataset. For ATAC-seq, three sources were used, GTRD (27, 28), ATACdb and ENCODE. For ATACdb, precision scores were determined at various p-value thresholds. For ENCODE, narrow peaks with and without IDR thresholding were used to calculate precision score. The numbers indicate the number of unmerged genomic intervals for each feature summed over datasets. For consensus footprints, genomic intervals were determined as a consensus across biosources using a posterior probability > 0.99 (63, 64). For the combined footprint and eRNA, the number represents the merged and intersecting genomic intervals.

Figure S4

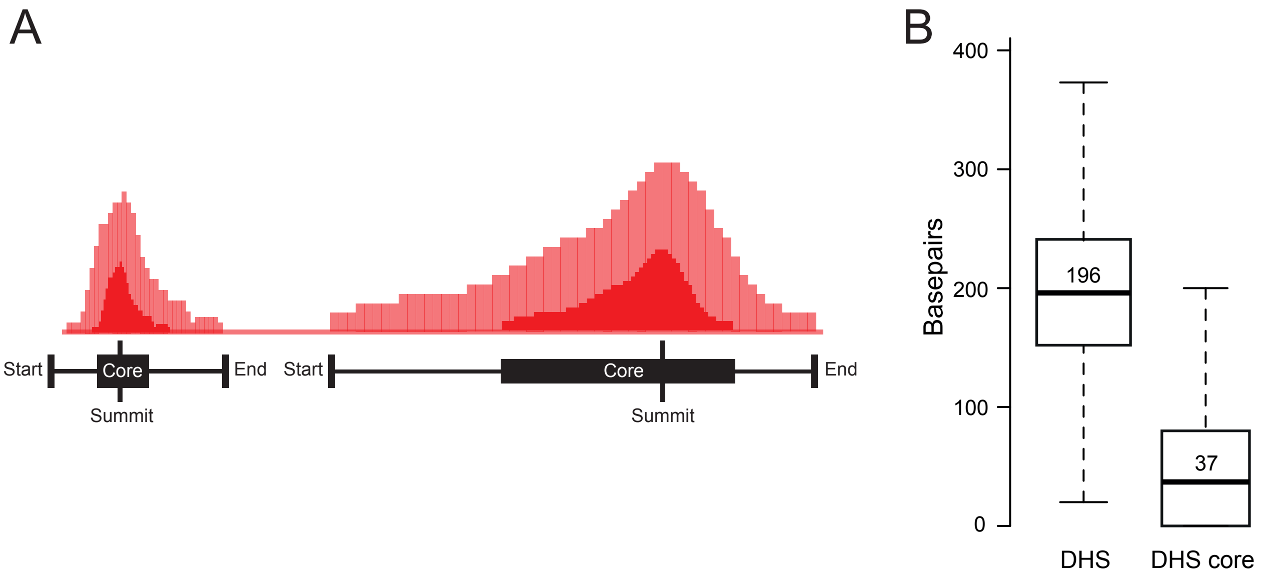
A
Start
Core
End
Start
Core
End
Summit
Summit
B
400
300
200
100
0
Basepairs
196
37
DHS
DHS core

Figure S4. Features of DHS centrality measures.

A. Schematic of DNase hotspots showing summits, DHS cores, and start and end locations.

B. Boxplot of unmerged DHS lengths compared to DHS cores.

Table S1

Trait	No of studies	Variants Total	Variants Non-redundant	Variants in FP	Variants in eRNA	Variants in FP/eRNA
Leukocyte count (WBC)	51	19264	10158	722	1501	333
Body Height (exc Child traits)	53	7487	6038	341	653	141
Body Mass Index (BMI) (exc Child traits)	114	8672	4922	195	344	56
HDL Cholesterol (HDL)	123	10872	3969	200	503	78
Bone density (BD)	63	5883	3755	190	351	63
Triglycerides (TRIG)	114	8567	3737	184	423	72
Type 2 diabetes (T2DM)	133	5679	3612	172	326	59
Systolic blood pressure (SBP)	73	4227	3096	173	341	73
FEV1:FVC ratio	23	3283	2957	121	165	36
Mean corpuscular haemoglobin (MCV)	29	4206	2779	201	508	121
Platelet count (PLT)	28	3686	2545	187	387	89
Glomerular filtration rate (GFR)	30	3726	2442	159	287	68
Alzheimer's diseae (AD)	88	2811	2380	115	231	1
Monocyte count (MC)	20	3474	2300	179	375	83
Asthma	93	3442	2275	116	286	65
Diastolic blood pressure (DBP)	69	3031	2203	106	223	43
Coronary artery disaese (CAD)	72	3037	2062	119	203	47
Breast carcinoma (BC)	100	2566	1852	66	163	20
Mean platelet volume (MPV)	17	2046	1423	104	283	64
Inflammatory bowel disease (IBD)	78	2075	1134	58	148	29
Rheumatoid arthritis (RA)	69	1760	1094	70	160	40
Prostate carcinoma (PC)	57	1577	948	61	107	31
Systemic lupus erythromatosis (SLE)	41	1197	936	49	145	27
Vital Capacity (VC)	17	916	844	0	0	0
Lung carcinoma (LC)	55	1058	823	24	73	15
Crohn's Disease (CD)	45	995	747	43	96	20
Multiple sclerosis (MS)	41	819	727	48	123	23
Psoriasis	28	895	691	44	98	20
Chronic Kidney Disease (CKD)	40	1111	689	33	63	13
Type 1 diabetes (T1DM)	41	815	658	28	83	18
Heart rate (HR)	27	792	582	22	41	10
Age at menarche (Men)	16	636	579	27	35	6
Cholesterol:total lipids ratio (Chol) (exc Child traits)	2	1054	568	36	89	14
Ulcerative colitis (UC)	28	753	556	33	84	17
Atrial fibrillation (AF)	20	767	477	32	50	11
Stroke	30	618	477	19	31	4
Ovarian carcinoma (OC)	19	600	448	19	24	5
Parkinson's Disease (PD)	50	546	434	19	42	7
Melanoma	26	519	392	23	33	8
Eczema	4	505	391	28	69	16
Ankylosing spondylitis (AS)	9	416	370	25	50	13
Primary sclerosing cholangitis (PSC)	6	299	282	26	43	15
Hypothyroidism	7	292	259	17	35	7
Age at menopause (AAM)	11	335	254	12	40	5
Amytotrophic lateral sclerosis (ALS)	27	262	223	12	13	3
Celiac Disease (CEL)	13	249	218	10	27	3
Primary biliary cirrhosis (PBC)	8	252	196	12	39	5
Juvenile idiopathic arthritis (JIA)	11	148	145	3	15	1
Colorectal carcinoma (CC)	7	129	127	8	12	3
Idiopathic pulmonary fibrosis (IPF)	6	70	58	7	16	4
Glioma	9	153	54	4	8	2
Renal carcinoma (RC)	7	50	39	2	4	0
Narcolepsy (NAR)	8	36	31	1	5	0

Table S1. Co-localisation of GWAS traits from the GWAS catalogue with DNase footprints and eRNA.

53 GWAS traits from the GWAS catalogue, showing the number of studies, total number of variants, and number of non-redundant variants. Non-redundant variants were then intersected with DNase footprints, eRNA or both. The numbers co-localising are shown and the table sorted by non-redundant variant number.

Table S2

GWAS Trait	GWAS variants	LD Variants	Non-Redundant Variants (GWAS and LD)	DHS	Footprints	ATAC (GTRD)	ATAC (Encode+IDR)	H3K27ac	ChIP	eRNA	Footprint + eRNA
Age at menopause	689	27981	20716	5096	615	17987	3234	15394	6113	1011	192
Age at menarche	254	13830	10977	2614	371	9543	6005	8923	3495	801	144
Alzheimer's diseae (AD)	2310	70728	46412	10893	1366	39800	15202	34024	13005	2659	466
Amytotrophic lateral sclerosis (ALS)	221	7073	6224	1336	157	5299	1382	4420	1627	274	40
Ankylosing spondylitis (AS)	369	12098	10635	2980	482	9341	3749	8634	3611	899	184
Asthma	2169	69240	31400	8520	1035	27070	10059	24526	9362	2428	424
Atrial fibrillation (AF)	476	17176	10143	2823	391	8944	3090	8476	3234	678	120
Body Height (exc Child traits)	578	206211	133969	34417	4612	116535	43673	105839	41543	8259	1523
Body Mass Index (BMI) (exc Child traits)	4903	193051	107270	24312	2908	93466	31165	78034	28949	4846	870
Bone density (BD)	3739	122864	71425	18716	2676	62679	23717	57656	22332	4299	862
Breast carcinoma (BC)	1483	47713	26034	6767	839	22666	9113	20691	8147	1507	260
Celiac Disease (CEL)	218	7645	4966	1409	216	4377	1695	4083	1761	447	82
Cholesterol:total lipids ratio (Chol) (exc Child traits)	654	13946	9256	2675	339	8063	3246	7542	3026	765	113
Chronic Kidney Disease (CKD)	568	25229	15542	3369	424	13181	3952	10681	3993	773	145
Colorectal carcinoma (CC)	127	2187	1620	365	47	1421	365	1228	450	99	20
Coronary artery disaese (CAD)	2046	81289	35025	9245	1303	30551	11772	27888	10889	2247	409
Crohn's Disease (CD)	743	29773	18137	5111	778	15850	6119	14740	5927	1630	310
Diastolic blood pressure (DBP)	2150	102257	52966	13494	1798	45972	16751	41236	16283	3426	652
Eczema	391	12519	9339	2735	387	8145	3130	7706	3070	852	155
FEV1:FVC ratio	2913	92912	34796	9162	1252	30465	11453	27505	10464	1914	358
Glioma	54	1620	1201	345	50	1013	420	942	385	118	26
Glomerular filtration rate (GFR)	2437	100205	54105	13930	2011	46839	18032	43144	17220	3790	747
HDL Cholesterol (HDL)	3737	124638	58260	15086	2114	50100	20608	45387	18070	4186	767
Heart rate (HR)	6014	16424	12271	2979	391	10645	3541	8835	3271	720	115
Hypothyroidism	257	7126	5973	1781	268	5280	2372	5188	2076	549	92
Idiopathic pulmonary fibrosis (IPF)	43	3853	3597	1138	124	3148	1277	3078	1371	185	38
Inflammatory bowel disease (IBD)	1105	44734	24368	6872	989	21227	8238	19498	7873	2097	394
Juvenile idiopathic arthritis (JIA)	145	4887	4142	1076	145	3590	1445	3191	1317	318	58
Leukocyte count (WBC)	9997	344973	144755	39786	5904	125680	47486	118877	48727	12336	2383
Lung carcinoma (LC)	814	24660	13674	3510	464	11780	4857	10599	4186	1020	204
Mean corpuscular haemoglobin (MCV)	2251	89214	47946	13053	1938	41463	15477	39758	16484	4124	833
Mean platelet volume (MPV)	389	42938	24079	7020	1037	20991	8559	20300	8459	2398	428
Melanoma	1380	11157	7651	1988	267	6657	2108	6128	2314	433	94
Monocyte count (MC)	2746	151105	79242	22100	3262	68672	26123	66208	27546	6861	1362
Multiple sclerosis (MS)	578	20793	14798	4634	644	12773	5018	12502	5174	1524	288
Narcolepsy (NAR)	31	1147	1156	411	39	901	318	949	318	204	24
Ovarian carcinoma (OC)	404	11959	8645	2290	273	7480	2572	6568	2755	435	81
Parkinson's Disease (PD)	419	473	882	264	34	778	275	671	279	66	12
Platelet count (PLT)	2515	81102	43112	12132	1838	37434	14530	36178	14734	3772	700
Primary biliary cirrhosis (PBC)	195	8753	6412	2050	240	5587	2162	5463	2329	511	89
Primary sclerosing cholangitis (PSC)	282	9338	9475	2890	461	8372	3436	7941	3379	866	180
Prostate carcinoma (PC)	933	26643	18425	4608	614	15820	5646	14071	5693	1152	202
Psoriasis	676	20588	15475	4348	702	13547	5342	12727	5381	1370	295
Renal carcinoma (RC)	38	1198	1117	278	38	945	335	838	367	87	17
Rheumatoid arthritis (RA)	1088	32983	22571	6301	879	19703	7595	17855	7341	1784	340
Stroke	470	12299	10239	2643	348	9009	3220	8092	3128	630	93
Systemic lupus erythromatosis (SLE)	921	32685	22840	5690	794	19521	6660	17291	6725	2057	343
Systolic blood pressure (SBP)	3038	133330	70910	17889	2416	61579	22974	54818	21558	4266	830
Triglycerides (TRIG)	3478	111128	57602	14585	2002	49570	19099	43863	17375	3810	730
Type 1 diabetes (T1DM)	445	15598	13219	3813	496	11513	4397	10645	4357	1108	185
Type 2 diabetes (T2DM)	3322	116693	67230	16555	2071	58132	22239	51065	19643	3631	672
Ulcerative colitis (UC)	556	21547	15299	4259	641	12770	5326	11869	4875	1395	265
Vital Capacity (VC)	811	44973	32182	8107	1039	27262	10240	24625	9627	1677	312

Table S2. Co-localisation of GWAS variants and linkage disequilibrium (LD) variants with genomic features of active regulatory element.

Variants from 53 GWAS traits from the GWAS catalogue were analysed for variants in LD with each GWAS variant. The combined set of GWAS variants and all LD variants for each trait were intersected with DHS (DHS from DNase-seq), DNase footprints, ATAC-seq (from GTRD, and ENCODE with IDR (irreproducible discovery rate) thresholding), H3K27ac, chromatin immunoprecipitation (ChIP), eRNA, and the combination of footpritns and eRNA.

Table S3

Dataset	Source	Version	Website	Genome build
DHS	ENCODE	Nov 23, 2021	https://www.meuleman.org/research/dhsindex/	Hg38
Footprints	ENCODE	OCT 20, 2020	https://www.vierstra.org/resources/dgf	Hg38
H3K27ac_GTRD	GTRD	v21.12	https://gtrd.biouml.org	Hg38
ATAC-seq	GTRD	v21.12	https://gtrd.biouml.org	Hg38
eRNA	PINTS	2021v1	https://pints.yulab.org	Hg38
ChIP-Seq	GTRD	v21.12	https://gtrd.biouml.org	Hg38
GWAS Catalog	NHGRI-EBI	V1.0.2	https://www.ebi.ac.uk/gwas/docs/file	Hg38
ChIP-Seq_ASB	AD_ASTRA	V4.0.3	https://adastra.autosome.org/zanthar	Hg38
H3K27ac_dbInDel	dbInDel	v2018	http://enhancer-indel.cam-su.org	Hg19
CAV	ENCODE	OCT 20, 2020	https://www.vierstra.org/resources/dgf	Hg38
raQTL_MPRA	Primary_paper	NA	https://osf.io/w5bzq/wiki/home/?view	Hg19
caQTL	QTLbase	v1.3	http://www.mulinlab.org/qtlbase/studies.html	Hg19
CRISPRdel_CRISPRi	Primary_paper	NA	https://www.nature.com/articles/s41587-022-01211-7#MOESM	Hg38
ATAC-seq	ATACdb	v1.03	https://bio.liclab.net/ATACdb/index.php	Hg38
ATAC-seq	ENCODE	v104	https://www.encodeproject.org	Hg19

Table S3. Data sources.

The table shows the source, version and genome build for each dataset. Where the genome version differs, data has been converted to Hg38 using Liftover (see methods).
