# Supplementary material for: DNA-binding factor footprints and enhancer RNAs identify functional non-coding genetic variants
Source: Genome Biol. 2024 Aug 6;25:208. doi: 10.1186/s13059-024-03352-1 (PMC11304670; doi:10.1186/s13059-024-03352-1)
Supplement: Supplementary file 2 — Additional file 2. Review history. [file 13059_2024_3352_MOESM2_ESM.docx]

Review History

**First round of review**

**Reviewer 1**

**Comments to author:**

Review Commons Report 1. <https://doi.org/10.15252/rc.2024235645>.

**Reviewer 2**

**Comments to author:**

Review Commons Report 2. <https://doi.org/10.15252/rc.2024765375>.

**Authors Response**

**Point-by-point responses to the reviewers’ comments:**

*We thank the reviewers for their helpful comments on our manuscript, which systemically analyses commonly used regulatory marks for their ability to uncover functional regulatory DNA. We report the precision for these marks, and uncover the combination of DNase footprints and eRNA as strong predictors of function. This has not been previously described. Furthermore, our systematic approach covers common trait- and rare disease-associated variants.*

*The reviewers raised points that we have addressed, which improves our manuscript. Some comments are similar between the two reviewers. Addressing these has increased the rigor of the manuscript and provides clear recommendations for the prioritization of functional variants.*

**Responses to Reviewer 1**

1. The authors should expand the QTL studies and the GWAS variants via LD and recompute the enrichments. For example, I would take all variants in high LD (r2 > 0.8 or 0.9) with either a QTL or GWA-variant. This will likely increase total variants overlapping an annotation, but reduce overall enrichment (odds-score), and possibly provide some information about which chromatin marks are more associated with "causal" variants.

*The reviewer highlights an important point that lead variants from GWAS are not often the causal variant. We highlight this in our manuscript for one variant, but as the reviewer states, an expanded analysis for LD will likely be informative.*

*We therefore expanded our analysis of variant intersections with genomic features by including variants in LD with GWAS variants from the 53 traits we have analyzed. We used an r2 > 0.7 as this cutoff is consistent with the example provided in our manuscript, but also because r2 higher than this is likely to be too stringent. We analyzed the LD variants to generate precision and recall scores for each trait and determine the mean precision and recall score for each feature (Figure 6A, and 6B). Our findings are consistent with our analysis of rare, non-coding variants, showing the combined footprints and eRNA have the highest precision, but with low recall. We also report the number of variants that intersect with feature(s) (Supplementary Table 2). Together, this provides important information to validate our approach, strengthens the section on LD, and provide readers with information on how considering variants in LD impacts prioritization. We discuss this analysis from Line 399.*

2. Can the authors comment on why eRNAs seem to be such a strong marker of functional variation? Are these just "strongest" (most accessible) distal elements? I would assume that these peaks have high overlap with chromatin accessibility peaks.

*We have added a section in the discussion to expand on eRNAs (Line 464). Here we refer to reports using eRNAs to predict TF binding (Azofeifa et al. 2018, Genome Res. 28, 334), where eRNA co-expression marks enhancer-promoter interactions (Lee at al., 2023, Scientific Reports. 23:19085), and for eRNA as independent predictors of functional enhancers in vivo (Wu et al., 2014, PLoS Genetics. 10:e1004610). These reports demonstrate that eRNAs are associated with active regulatory elements that are likely to drive gene expression. To corroborate this we asked if GWAS variants that overlap different features are associated with functional enhancers. Using a curated list of functional enhancers (Yao et al., 2022), we find that GWAS variants in footprints or eRNA are associated with functional enhancers compared to other marks, with their combination showing higher enrichment of functional enhancers (Supplementary figure 3B).*

*To address overlapping features, we determined which other active regulatory marks are associated with each other, including for eRNA using pair-wise analysis. We computed the Jaccard index to quantify intersection similarity between features, and observe the highest Jaccard index for ATAC and H3K27ac (Figure 1D). This is discussed on Line 149.*

3. Would the ATAC-seq enrichment increase if the authors stratified regions by signal rather than aggregating all peaks? The vast majority of chromatin accessibility peaks are very weak and could be false-positives. Lets imagine that in each dataset 1% of peaks are FPs and that the FP peaks are mostly randomly distributed accross the genome. As such, aggregating hundreds to thousands of samples would have many FP peaks and greatly affect the enrichment analysis. Conversely, DNase I footprints are found in high signal peaks that are less likely to be false-positives. One approach to deal with this is to select ATAC-seq peaks matched to the peak signal in DHS peaks with footprints.

*The reviewer raises an important point of noise-to-signal effects on false-positives, and that how a dataset is analyzed, and the significance or FDR cutoff, could impact precision for functional variants. This may impact the analysis for ATAC-seq, and other datasets. We used an independently analyzed set of ATAC-seq data (~1400 datasets) from ATACdb, where peaks were determined using an FDR < 0.05 (the same as the GTRD datasets we used in our manuscript). We calculated the precision score for this, against other feature(s), relative to precision score by GWAS alone (as fold change). We then threshold by three p-value cutoffs (0.001, 0.001, 0.0001) which are p-values for the signal over local background. Importantly, different dataset sources can alter the precision score, but increasing stringency has minor effects. We also tested the peak calling approaches by using a third independent analysis for ATAC-seq, from ENCODE, using MACS2 narrow peak calling of ATAC-seq data, and narrow peaks thresholded by irreproducibility discovery rate (IDR), and compared the precision scores across the ATAC-seq analyses. Thresholding by IDR for the ENCODE data improves precision score (Supplementary figure 3C). We have avoided matching ATAC-seq with DNase-seq peak however as suggested by the reviewer. We prefer the above approach as this maintains independence between ATAC-seq and DNase-seq, minimizing cross method bias.*

*Other markers may also be impacted by stringency during calling. The reviewer comments that footprints are more likely to be found in high signal peaks, but footprints are found in smaller DNase peaks (Vierstra et al., 2020 dataset used in our manuscript). Nevertheless, the footprint set we have analyzed utilized a consensus analysis across datasets using a posterior probability > 0.99 (Vierstra et al., 2020). We expanded our analysis and compared footprints from each biosource using FDR cutoffs at < 0.01 and < 0.001 to < 0.05 and compute precision scores to determine if footprint calling stringency alters precision score. Increasing footprint calling stringency using FDR increases precision scores, but the consensus set we have used throughout provides a high precision score (Supplementary figure 3C). We discuss the importance of thresholding calling methods from Line 279 as a new section*.

Minor comments

Figure 1c -- no legend is provided specifying what the bar colors represent.

*This has been corrected.*

Pg. 10 -- "To overcome this, detection of footprints could be improved by enzymatic digestion bias correction (Calviello et al., 2019)." The DNase I footprinting dataset used in this paper performs extensive bias correction using a 6mer statistical model. Nevertheless, I completely agree the sentiment of the authors that low and variable sensitivity of footprinting is certainly driving a high false-negative rate with regards to comprehensively identifying function variants.

*We have improved the accuracy of this statement by making “bias correct” more general.*

Figure 3 is a little too complicated for its purpose, which is to show the enrichment of bQTLS, caQTLs and raQTLs.

*We have simplifed this figure by altering the presentation and changing pie charts in 3B to bar graphs.*

**Responses to Reviewer 2**

1. I found this line in the abstract unclear: "This signature provides high precision, trading-off low recall".

*We have made this statement clearer to now read “This signature provides high precision, but with a trade-off of low recall”.*

2. Figure 1C is missing a y-axis label and a colour legend.

*This has been corrected.*

3. The authors note a high genomic coverage "by ATAC-seq (75.2%), [and] H3K27ac (61.5%)", which they attribute in part to "too low a threshold used in peak calling". However, both the benchmark datasets and the genomic predictors are utilized without consideration of the effect of thresholding. To what extent are DNase footprints and eRNA specifically informative, vs. representing datasets processed with highly selective cutoffs?

*This comment is similar to Point 3 raised by Reviewer 1. We have analysed thresholds for both footprints and ATAC-seq and presented this in Supplementary figure 3C and discussed this as a new section (Line 279).*

4. The benchmark datasets are described as molQTL, bWTL, and caQTL. "QTL" implies a regression of a trait (e.g. accessibility) on a genotype. This is not accurate here: the Vierstra and Abramov datasets investigate allelic imbalance (a related but orthogonal approach), while van Arensbergen is an MPRA.

*The reviewer raises an important point of nomenclature. The field is inconsistent in this regard, which we accept can be challenging for readers. For example, variants that have effects on chromatin accessibility have been reported as caQTL but also CAV (chromatin-altering variants). For TF binding, this has been reported as binding QTL (bQTL), but also ASB (allele specific binding). For reporter assays, this has been reported as raQTL (as in van Arensbergen et al), or elsewhere as allele-specific activity or allele-specific* *expression. To minimize the burden of various abbreviations, we have chosen to unify this using the QTL terminology, as allele-specific imbalance described as QTLs have been accepted for many features including those used in our manuscript. We have included a statement in Line 163 to clarify this.*

5. QTLbase is cited alongside the Vierstra paper. I had some trouble searching in QTLbase but did not find the Vierstra dataset. It should be clarified whether QTLbase was used to download the Vierstra results, or to supplement it with other studies.

*The caQTL variants were obtained from both QTLbase (called caQTL) and Vierstra et al (called CAV - chromatin-altering variants in their paper), and merged. On Line 184, we now state that caQTL are derived from two sources.*

6. Fig. 4 discusses the effect of variant centrality in a DHS for prioritization, but it isn't included in the FINDER schematic in Fig. 7. How come it wasn't employed in FINDER?

*We observed that feature centrality imparts small improvements in precision scores for some marks such as DNase-seq. However, this is poor compared to footprints and eRNA combined without regard to centrality. Therefore, we do not implement centrality in the FINDER framework. We have clarified this in the discussion (Line 456).*

7. The Discussion notes "Firstly, the identification of DNase footprints may be related to residency time of TFs on DNA, where rapidly exchanging factors impart poor footprints (Sung et al., 2014). Variants associated with altering binding of dynamic factors may therefore be missed. To overcome this, detection of footprints could be improved by enzymatic digestion bias correction". I don't see how enzymatic digestion bias is related to sensitivity to detect rapidly exchanging TFs.

*Reviewers 1 and 2 raise similar comments. We have clarifed this statement and added a reference which describes a method using crosslinking with DNase-seq for footprint detection of rapidly exchanging transcription factors (Oh et al., 2019, Epigenetics chromatin. 12:30).*

8. It looks like the eRNA data were obtained from GRO-seq or PRO-seq data. It would be helpful to note key details like this directly rather than leaving it to the reader to try to figure out what is in the PINTS database.

*We have included a statement that lists some of the experimental methods used to determine the eRNA data in PINTS, which includes PRO-seq, GRO-seq, CAGE-seq and others (Line 138). We have commented on which contributes most to the dataset, but also in the discussion we comment on which particular assay for eRNA is recommended based on previously published data (Line 515).*

9. The github link https://github.com/sbiddie/FINDER gives a 404 not found error. Is FINDER an actual tool implementation, or more generally describing the approach?

*The github link for the FINDER framework consists only of merged features of active regulatory elements. These can be used with Bedtools, enabling others to prioritize variants in a computationally inexpensive way. The github link will be released upon final publication of the manuscript.*

10. Some of the figure text is quite small or blurry (e.g. Fig. 1A/B, Fig. S2).

*We have rectified this for all figures.*

11. Typo in Figure 1A legend: "Heatpmap".

*This has been corrected.*

12. The author of the last entry in the References is "Zhen Z" but should be "Zheng Z".

*This has been corrected.*

**Second round of review**

**Reviewer 2**

The authors have addressed the reviewer comments. Congratulations on the nice manuscript!
